# Temperature Prediction Based on Bidirectional Long Short-Term Memory and Convolutional Neural Network Combining Observed and Numerical Forecast Data

**DOI:** 10.3390/s21030941

**Published:** 2021-01-31

**Authors:** Seongyoep Jeong, Inyoung Park, Hyun Soo Kim, Chul Han Song, Hong Kook Kim

**Affiliations:** 1 Gwangju Institute of Science and Technology, School of Electrical Engineering and Computer Science, Gwangju 61005, Korea; wp030700@gist.ac.kr; 2 Gwangju Institute of Science and Technology, AI Graduate School, Gwangju 61005, Korea; pinyoung@gist.ac.kr; 3 Gwangju Institute of Science and Technology, School of Earth Sciences and Environmental Engineering, Gwangju 61005, Korea; hskim98@gist.ac.kr (H.S.K.); chsong@gist.ac.kr (C.H.S.)

**Keywords:** temperature prediction, bidirectional long short-term memory (BLSTM), convolutional neural network (CNN), regional data assimilation and prediction system, automatic weather station, weather data

## Abstract

Weather is affected by a complex interplay of factors, including topography, location, and time. For the prediction of temperature in Korea, it is necessary to use data from multiple regions. To this end, we investigate the use of deep neural-network-based temperature prediction model time-series weather data obtained from an automatic weather station and image data from a regional data assimilation and prediction system (RDAPS). To accommodate such different types of data into a single model, a bidirectional long short-term memory (BLSTM) model and a convolutional neural network (CNN) model are chosen to represent the features from the time-series observed data and the RDAPS image data. The two types of features are combined to produce temperature predictions for up to 14 days in the future. The performance of the proposed temperature prediction model is evaluated by objective measures, including the root mean squared error and mean bias error. The experiments demonstrated that the proposed model combining both the observed and RDAPS image data is better in all performance measures for all prediction periods compared with the BLSTM-based model using observed data and the CNN-BLSTM-based model using RDAPS image data alone.

## 1. Introduction

Since the beginning of human history, human beings have experienced various weather and climate changes, some of which have driven them to change their place of residence. Weather change may cause the rise of sea levels by melting glaciers, and extreme weather events, such as heat waves and torrential downpours, are becoming more intense and frequent. These changes not only devastate the environment, but also restrict human activities, even placing human lives in danger [1]. Over the past decade, weather change has been accelerating, and many studies have been conducted to analyze and develop responses to the causes of the change [2].

Recently, deep learning and machine learning have been widely used as modeling methods for predicting future data, such as air quality, wind speed, and power demand [3,4,5]. Deep learning is capable of handling complex nonlinear relationships. In particular, when processing time series data, recurrent neural networks (RNNs), including long short-term memory (LSTM), are often used and have been shown to have better performance compared with other conventional methods [6]. While various studies in Korea are conducted using meteorological information collected by the Meteorological Agency, these data inevitably contain flaws, due to sensor and communication issues.

For example, the authors of a recent study [7] pointed out a problem with poor prediction accuracy due to missing observed data from sensors installed at the observatory. To solve this problem, a deep learning-based refinement model was proposed in [7], and the prediction model using the refined data provided better prediction accuracy than the model using data approximated using linear interpolation. The accuracy of future weather prediction can also be increased by augmenting the data using satellite information or combining a variety of types of information [8,9].

To predict weather information for a specific area, it is typical to construct a prediction model only using observed data from the automatic weather station (AWS) closest to the area. However, weather changes in a specific area are not affected solely by the geographical, spatial, and temporal factors of the area. Therefore, it should be possible to improve the accuracy of weather prediction for a specific area by combining the observed data from the areas near the specific area, rather than using only data from the area. There are several issues influencing how much of the neighboring area should be chosen for the modeling, as well as which weather factors from the nearby areas should be combined with those of the target area.

The relationship between weather factors in an area and the nearby areas can change over time, depending on the wind direction or seasonal factors. Instead of selecting weather factors or nearby areas, the weather data of the region covering the specific area can be combined with the weather factors from the target area. The first method of using regional data is to take the observed data from all the AWSs located in the region. In this case, it should be assumed for the prediction model that all AWS data should include no errors or missed data, which is a situation that is not guaranteed in practice. Recently, there have been several studies indicating that combining numerical forecast data with observed data improved the accuracy of temperature prediction [9] and aerosol prediction [10].

On the basis of this research, we incorporate numerically forecast data into a temperature prediction model. In particular, a regional data assimilation and prediction system (RDAPS) provides forecast data in the form of images [11]. The proposed temperature prediction model applies two different deep neural networks, RNNs and convolutional neural networks (CNNs), to the observed time-series data of an AWS and the numerally forecast image data, respectively. In the case of image data, the outputs of the CNN are further processed by another RNN.

After that, the output layers of the neural networks are concatenated and further processed by a dense layer to predict the temperature. Temperature prediction is carried out up to 336 h (14 days) in advance, with weather data for up to 24 h as input. The proposed model also provides 6-, 12-, 24-, 72-, and 168-h (7-day) temperature predictions. The performances of the proposed temperature prediction model are evaluated by measuring the index of agreement (IOA), Pearson correlation coefficient (R), root mean squared error (RMSE), mean absolute error (MAE), mean bias error (MBE), mean normalized gross error (MNGE), and mean normalized bias (MNB) between the real observed temperatures and those predicted by the proposed model.

The remainder of this paper is organized as follows. In Section 2, we discuss the methodology of deep learning-based temperature prediction models using weather data from the Korean Meteorological Agency. Then, in Section 3, we propose a hybrid temperature prediction model using the observed and RDAPS data. In Section 4, we report on the performance of the proposed model and compare the results with other deep learning models. Finally, in Section 5, we summarize and conclude the paper.

## 2. Related Work

The Korean Meteorological Agency (KMA) has been producing weather forecasts using the British Meteorological Agency Model (UM) since 2010 [12]. The weather forecast system mainly consists of three numerical weather prediction (NWP) models: a global data assimilation and prediction system (GDAPS), an RDAPS, and a local data assimilation and prediction system (LDAPS). The RDAPS forecasts provided by the KMA cover the East Asian region, and they are good at predicting the weather on average. However, the prediction accuracy depends on the geographical properties of the area covered. Thus, the prediction accuracy of the RDAPS is limited when the area has complex topography, like the Taebaek Mountains and the East Sea, which remains a challenge to NWP models [13].

To accommodate the dynamic behavior of weather changes described above, different types of weather data can be integrated using a machine learning or deep learning framework [14,15,16]. For example, an experiment for the prediction of aerosols was conducted using two different types of data, one type from a chemical forecast model for predicting pollutants that could affect the concentration of aerosols, and the other the aerosol data themselves [16]. A machine learning approach based on extreme gradient boosting, and a light gradient boosting machine, as well as a deep learning approach based on LSTM were used to predict particulate matter 10 or 2.5 (PM_10_ or PM_2.5_) by combining the observed data and data produced by the Community Multi-scale Air Quality (CMAQ) model [10].

This approach produced better prediction accuracy for the PM_10_ and PM_2.5_ levels than those only using the observed data. However, the observed data are time series, but the CMAQ model data are two-dimensional (2D). This discrepancy in the format was overcome by converting the 2D CMAQ model data into a one-dimensional (1D) time-series prior to using the machine learning and deep learning models. As another example of combining different forms of data, the soil moisture sensing data and digital elevation model data were combined into a 2D format to predict the soil moisture with the combined data input to a neural network [17].

To deal with the different dimensionality of data according to the sources, such as observed or numerical data, we preserve each dimension of the different forms of data, instead of converting the dimension from 1D to 2D or vice versa. In particular, we apply CNNs and LSTMs to the observed 1D data from an AWS and the 2D image data from the RDAPS. This is because an LSTM can reflect the weather change over time for the 1D observed data, while a CNN can provide a feature map representing regional change over a given time, which is further processed by an LSTM to extract features over time for the RDASP image data.

An LSTM processes time-series data only in the forward direction. However, if the time-series data are somewhat periodic in time, processing in both the forward and backward direction may help improve feature representation, which results in a bidirectional long short-term memory (BLSTM) by combining a forward LSTM and a backward LSTM [18]. Due to the periodicity of temperature every 24 h, a BLSTM neural network is used in this paper instead of an LSTM, and we expect a BLSTM to provide better prediction performance than LSTM, as in other applications using time-series data [19,20]. Then, the two different feature layers are combined using a concatenation layer, which is fed into a temperature prediction layer.

## 3. Proposed Temperature Prediction Model Combining Observed and Numerical Forecast Data

**Figure 1:**
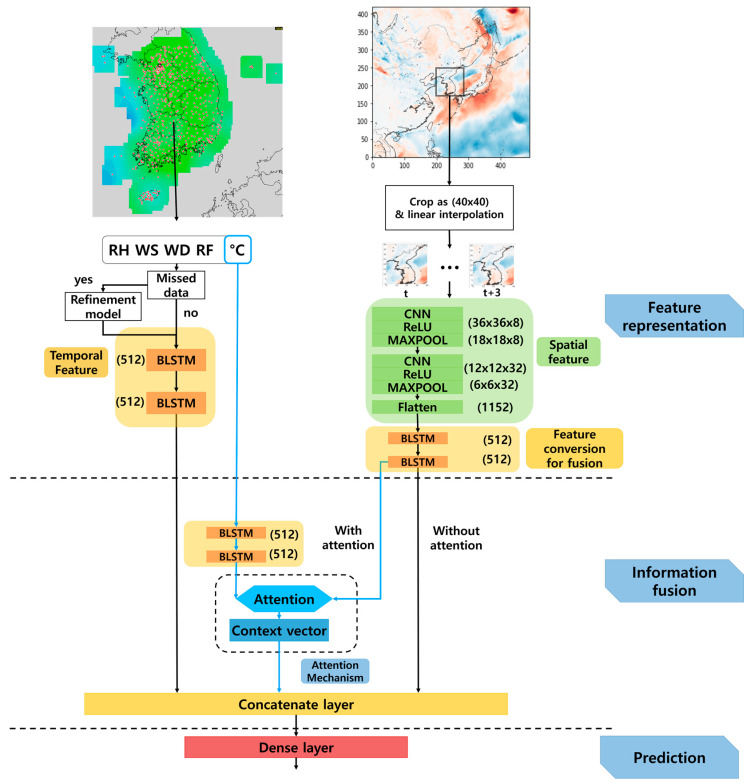
Block diagram of the proposed temperature prediction model composed of feature representation, information fusion, and prediction.

This section describes the deep neural-network-based temperature prediction model. As shown in Figure 1, the proposed model consists of three parts: feature representation, information fusion, and prediction. To train the model, two data sets are prepared. One is a set of AWS-observed data provided by KMA [21], in which the observed data are grouped into a five-dimensional vector at one-hour intervals, including the relative humidity (RH), wind speed (WS), wind direction (WD), rainfall (RF), and temperature in degrees Celsius (°C).

The other data set is numerical forecast data provided by the RDAPS from the KMA, where the temperature data for each three-hour interval are represented by a (491 × 419)-dimensional image. To predict the temperature in a specific area in the Korean Peninsula, the original image is cropped into a (40 × 40)-sized image, as shown in the upper right of Figure 1. A cropped image from a given time is interpolated by a factor of three to synchronize with the one-hour time interval used in the observed data.

In the feature representation part of the figure, two different neural networks are applied to combine the different types of input data. One is a BLSTM neural network to deal with the time-series observed data, and the other is a CNN for (40 × 40)-dimensional image data, followed by a BLSTM to deal with the multiple-hour image data. This network architecture is referred to as CNN-BLSTM feature representation. The former approach provided a temporal feature map, and the latter, a spatial feature map. The information fusion part of the proposed model combines two feature maps using a concatenation layer.

To emphasize the correlation of temperature between the observed and the numerical forecast image data, an attention mechanism [22] is applied prior to concatenating the feature maps. Finally, the prediction part is composed of a dense layer to predict the future temperatures by using the output of the information fusion part, where the mean squared error (MSE) between the target temperature and the predicted temperature by the dense layer is used as a loss function. The following subsections provide more detailed explanations on the data sets and each of three processing parts of the proposed temperature prediction model.

### 3.1. Data Sets

As mentioned earlier, two data sets are used in this work: AWS observations and the RDAPS data provided by KMA.

#### 3.1.1. Observed Data

The KMA provides a set of observed data once every hour for each of the 510 locations in Korea. The data include temperature, relative humidity, wind speed, wind direction, and precipitation. The observed data were collected over five years, from 1 May 2011 to 31 December 2015, and were divided into two datasets: the data from the four years from 1 May 2011 to 31 December 2014 were used as a training set, and the data for one year from 1 January 2015 to 31 December 2015 were used as an evaluation set.

The training set was further divided into two subsets that were 85% and 15% of the training data for the neural network model training and the validation of a trained model, respectively. Specifically, the observed data from 1 May 2011 to 15 May 2015 were used for training the models, and the remaining data from the training set were used for validating them for each epoch of the training. No cross validation is used in this paper. The period of observed data for the training set did not overlap with that for the evaluation data. Therefore, all the prediction models in this paper were trained and evaluated using the training data and evaluation data, respectively.

The problem of missing data is challenging when training deep learning models. In this work, the missing data from the observed data are refined by following the procedure described in [7]. Whenever any one element of the five-dimensional observed data at a given time is missing, all elements of the observed data are first refined by linear interpolation using two good data points from the previous time and the next time. The refined data are used for training the BLSTM model, and then the missing data are refined again using the trained BLSTM model.

#### 3.1.2. Numerical Forecast Data

As mentioned in Section 2, the weather forecasting system of KMA consists of three NWP systems: GDAPS, RDAPS, and LDAPS. The predictions of GDAPS and RDAPS are used as the boundary conditions for the operations of RDAPS and LDAPS, and their domains are represented in Table 1. As shown in the table, RDPAS and LDAPS cover East Asia and South Korea with horizontal resolutions of 12 × 12 km and 1.5 × 1.5 km, respectively. Both systems have 70 sigma vertical layers, but the top heights are set to 80 and 40 km for the RDAPS and LDAPS, respectively.

In addition to the observed data described in Section 3.1.1, the model proposed in this paper also uses numerical forecast data provided by the RDAPS. The RDAPS image data also provide information on the 70th floor in the vertical direction, which is the closest floor to the ground [23]. Since the RDAPS image data cover not only the Korean Peninsula but also other countries, such as China, Russia, and Japan, a part of the RDAPS data, which represents only the Korean Peninsula, are taken; thus, the (491 × 419)-sized image data are cropped into (40 × 40)-sized image data.

Prior to combining the RDAPS image data with the observed data, as discussed in Section 3.1.1, two preprocessing steps are performed. The KMA provides the RDAPS image data eight times per day, at 00:00, 03:00, 06:00, 09:00, 12:00, 15:00, 18:00, and 21:00 h; however, there exist missing RDAPS image data at certain operating times. Missing image data are refined by following the same technique used for the observed data, as explained in Section 3.1.1. Another preprocessing step for the RDAPS image data is to over-sample each image from a three-hour interval to a one-hour interval, because the time resolution of AWS observation is one hour. This preprocessing is simply performed using linear interpolation by a factor of three.

### 3.2. Feature Representation

The feature representation part of the proposed temperature prediction model is composed of two different types of neural networks to accommodate the different types of input data. These are a BLSTM neural network and a CNN to deal with the time-series observed data and the cropped RDAPS image data, respectively.

#### 3.2.1. BLSTM for Observed Data Representation

For the BLSTM-based feature representation from the observed data, the observed data are first normalized, because the dynamic range of the observed data differs from element to element, which might result in slow learning in a neural network. For example, the range of RH is from 0 to 100, and that of WD is from 0° to 360°. Among many data normalization approaches [24,25], a minimum–maximum (min–max) normalization technique is used in this work, which adjusts each element of the observed data from 0 to 1, using the following equation of

(1) $$x_{t,normal}=\frac{x_{t}-x_{min}}{x_{max}-x_{min}}$$

where $x_{t}$ is one of RH, WS, WD, RF, and °C at the *t*-th time, as shown in Figure 1. In addition, $x_{min}$ and $x_{max}\;$ are the minimum and maximum value of each element over the entire training set, and $x_{t,\;normal}$ is the normalized value at the *t*-th time.

**Figure 2:**
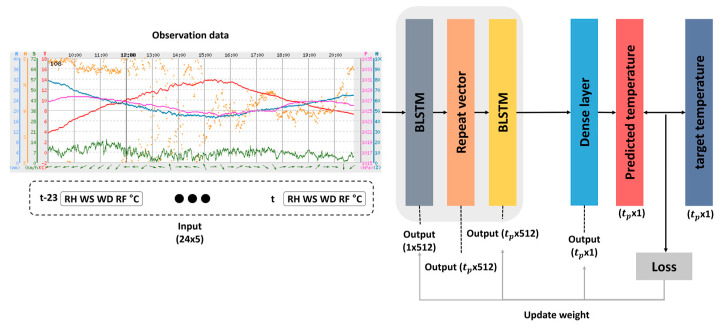
Block diagram of the bidirectional long short-term memory (BLSTM)-based feature representation module for the observed time-series data, where a dense layer works when only the BLSTM-based temperature prediction is performed.

The normalized data are then used as input features for the BLSTM-based feature representation module, as shown in Figure 2. For a given time, *t*, the observed data for up to 24 h from the past, from *t*-23 to *t*, are concatenated into a (24 × 5)-dimensional vector as the input feature. As shown in the figure, the feature representation module is based on a stacked BLSTM model [26] composed of two BLSTMs, a repeat vector layer, and a dense layer.

For a given (24 × 5)-dimensional input vector, the first BLSTM with 256 hidden nodes provides a 512-dimensional output, due to its forward and backward structure. Then, the 512-dimensional output from the BLSTM is input into a repeat vector layer that repeats the input vector $t_{p}$ times, resulting in a ($t_{p}$ × 512)-dimensional output vector. In this work, $t_{p}\;$ was the prediction time period for predicting the future temperature, and is set to one of 6, 12, 24, 72, 168, and 336 h.

The hidden vector of the last time step of the BLSTM is repeated instead of using the hidden vectors of all the time steps of the BLSTM, to diminish the risk of overfitting in the repeat vector layer [27]. Next, this ($t_{p}$ × 512)-dimensional vector is brought into the second BLSTM, in which the number of hidden nodes is also 256. Thus, the output of the second BLSTM is a ($t_{p}$ × 512)-dimensional vector, which is fed to the information fusion part to combine the features estimated from the RDAPS image data, as shown in Figure 1.

In parallel, the BLSTM-based temperature prediction is constructed by adding a dense layer that maps the ($t_{p}$ × 512)-dimensional vector into a ($t_{p}$ × 1)-dimension output vector. By doing this, the ($t_{p}$ × 1)-dimensional output vector from the dense layer can be compared with the target vector of the neural network that is also a ($t_{p}$ × 1)-dimensional vector for the future temperatures at the time period to be predicted, *t* + $t_{p}$. According to the MSE loss between the dense layer output and target vectors, the weights and biases of the stacked BLSTM are updated. The performance of this BLSTM-based temperature prediction only using observed data is discussed in Section 4.

#### 3.2.2. CNN-BLSTM for Numerical Forecast Data Representation

**Figure 3:**
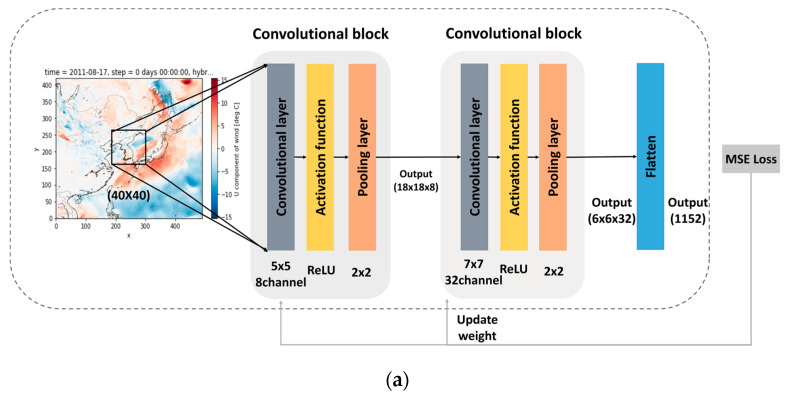

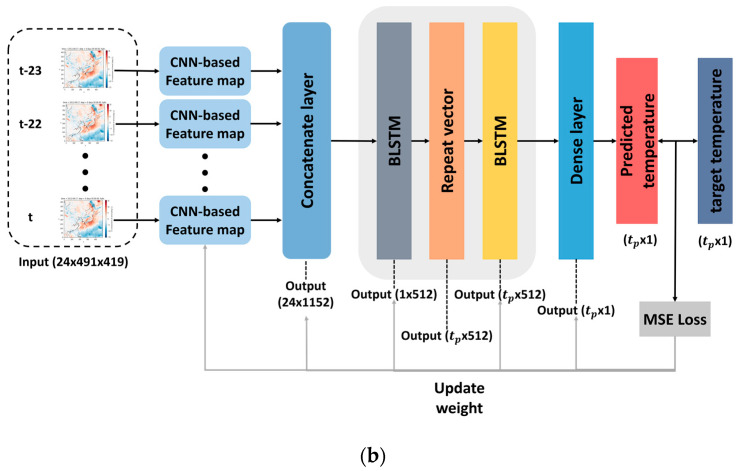
Block diagram of the convolutional neural networks (CNN)-BLSTM-based feature representation for regional data assimilation and prediction system (RDPAS) image data: (**a**) CNN-based feature representation for each time and (**b**) BLSTM–based feature representation over 24-h image data.

For the feature representation of the RDAPS image data, as described in Section 3.1.2, a CNN-based model is used to preserve the geographic and spatial information from the RDAPS image data [28]. Figure 3a shows a block diagram of the CNN-based feature representation module for the RDAPS image data, which consists of two convolutional blocks and a flattening layer. Each convolutional block is composed of a convolutional layer, a pooling layer, and an activation function. Each (40 × 40)-dimensional image, as described in Section 3.1.2, is used as an input to the first convolutional block, which consists of a convolutional layer, a rectified linear unit (ReLU) activation function, and a (2 × 2) max pooling layer, in which the convolutional layer has eight (5 × 5) kernels with a stride of (1 × 1).

The first convolutional block provides a (18 × 18 × 8)-dimensional feature map. This feature map is used as input to the second convolutional block. The convolutional layer of the second convolutional block has 32 (7 × 7) kernels. By processing an (18 × 18 × 8)-dimensional feature map using the second convolutional block, a (6 × 6 × 32)-dimensional feature map is obtained, and it is converted into a 1D feature by using a flattening layer, resulting in an 1152-dimensional feature for each input image.

After representing the image data as 1D data using the CNN-based feature representation module, temperature prediction is performed using a sequence of 24-h image data. The RDAPS image data are prepared once every hour, as mentioned in Section 3.1.2. Figure 3b shows a block diagram of BLSTM-based feature representation using the time-series data converted from the RDAPS image data, in which the network architecture of the BLSTM in Figure 3b is identical to that in Figure 2.

Each 1152-dimensional feature map is grouped into $t_{p}$ time-series feature vectors using the CNN-based feature representation, which is shown in Figure 3a. These feature vectors are then used as input features for the BLSTM feature representation module. Similar to the procedure described in Section 3.2.1, the MSE loss between the dense layer output and target vectors is calculated, and then the weights and biases of the stacked BLSTM and CNN are updated.

### 3.3. Feature Representation

**Figure 4:**
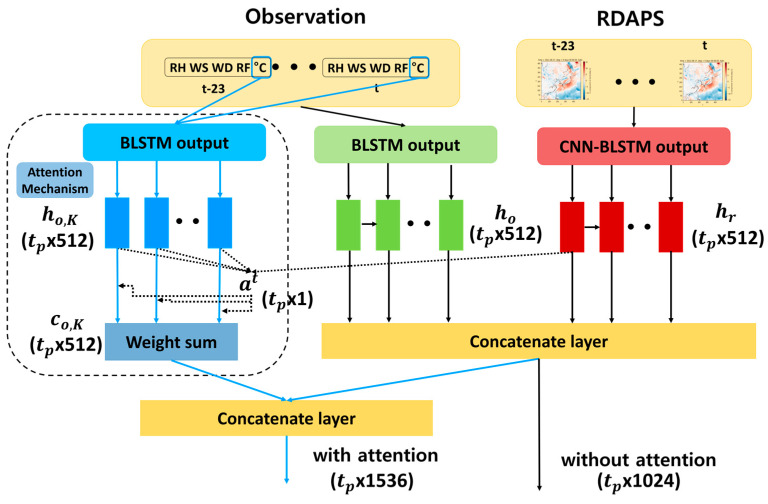
Block diagram of the information fusion part of the proposed temperature prediction model with and without an attention mechanism.

The information fusion part of the proposed temperature prediction model combines the feature vectors obtained from the BLSTMs applied to the observed data and those from the CNN-BLSTM applied to the RDAPS image data, as shown in Figure 1. As shown in the right part of Figure 4, denoted as without attention, the first information fusion is performed by combining all the hidden state outputs of the second BLSTM applied to observed data, $h_{o}=\left[h_{o}^{1},h_{o}^{2},\cdots ,h_{o}^{t_{p}}\right]$, and those of the second BLSTM applied to the RDAPS image data, $h_{r}=\left[h_{r}^{1},h_{r}^{2},\cdots ,h_{r}^{t_{p}}\right]$. Since $h_{o}^{t}$ and $h_{r}^{t}$ $\left(t=1,2,\cdots ,t_{p}\right)$ are all 1 × 512)-dimensional vectors, the dimension of the concatenated vector, $\left[h_{o};h_{r}\right],$ becomes ($t_{p}$ × 1024).

The second information fusion involves applying an attention mechanism [22] when combining the hidden states of both BLSTMs. As shown in the left part of Figure 4, in this information fusion, since the RDAPS image data are related only to temperature, while the observed data are composed of five different weather factors, only the temperature factor from the observed data is excerpted, and a BLSTM-based feature representation model is additionally constructed before applying the attention. In this case, the network architecture of the BLSTM used for temperature prediction is identical to the BLSTM shown in Figure 2.

By doing this, the ($t_{p}$ × 512)-dimensional hidden state output vector from the observed temperature data, $h_{o,K}=[h_{o,K}^{1},h_{o,K}^{2},\cdots ,h_{o,K}^{t_{p}}$], is used as a query and that from the RDAPS image data, $h_{r}^{t}$, as a key for the attention, where *t* ranges from 1 to $t_{p}$, as shown in the left part of Figure 4. Compared to the first information fusion, the attention mechanism aims to derive a context vector, $c_{o,K}=[c_{o,K}^{1},c_{o,K}^{2},\cdots ,c_{o,K}^{t_{p}}$], so that the relevant information of the RDAPS image data to the observed temperature data is exaggerated. Thus, instead of using $\left[h_{o};h_{r}\right]$ when an attention mechanism is not applied, $c_{o,K}$ is added to $\left[h_{o};h_{r}\right],$ producing a combined vector after applying the attention, $\left[h_{o};h_{r};c_{o,K}\right],$ with a dimensionality of ($t_{p}$ × 1536).

In this paper, the dot product attention is used to find $c_{o,K}$. To this end, the attention score function at the time step *t* is computed between all the hidden state outputs from the observed temperature data, $[h_{o,K}^{1},h_{o,K}^{2},\cdots ,h_{o,K}^{t_{p}}$] and the hidden state output at each time *t* from the RDAPS data, $h_{r}^{t}$, such as:

(2) $$score\left(h_{o,K}^{i},h_{r}^{t}\right)={\left(h_{o,K}^{i}\right)}^{T}h_{r}^{t},\;i=1,2,\cdots ,t_{p}$$

where *T* is the transpose operator. Then, a softmax function is applied to the scores for each time *t* to convert them into an attention distribution by using

(3) $$\alpha_{i}^{t}=\frac{\exp\left(score\left(h_{o,K}^{i},h_{r}^{t}\right)\right)}{\sum_{j=1}^{t_{p}}\exp\left(score\left(h_{o,K}^{j},h_{r}^{t}\right)\right)},\;i,t=1,2,\cdots ,t_{p}.$$

Next, $\alpha_{i}^{t}$ is multiplied to $h_{o,K}$ and summed up to $t_{p}$, such as:

(4) $$c_{o,K}^{t}=\sum_{i=1}^{t_{p}}\alpha_{j}^{t}h_{o,K}^{i},\;t=1,2,\cdots ,t_{p}.$$

Finally, $c_{o,K}$ is concatenated with $h_{r}$, resulting in $\left[h_{o};h_{r};c_{o,K}\right]$.

Lastly, the concatenated features are used for predicting the future temperature in the prediction part of the proposed temperature prediction model. This is performed using a dense layer, as described at the bottom of Figure 1. Since the target vector is given as a $(t_{p}$ × 1)-dimensional future temperature vector depending on the prediction period, $t_{p},\;$the number of the output units of the dense layer is $t_{p}$, where $t_{p}$ is one of 6, 12, 24, 72, 168, or 336 in this work. Eventually, the MSE between the target temperature and predicted temperature vector is calculated and this error is back-propagated to train all of the weights and biases of the neural networks employed in the proposed temperature model in Figure 1.

## 4. Experiments and Discussion

**Figure 5:**
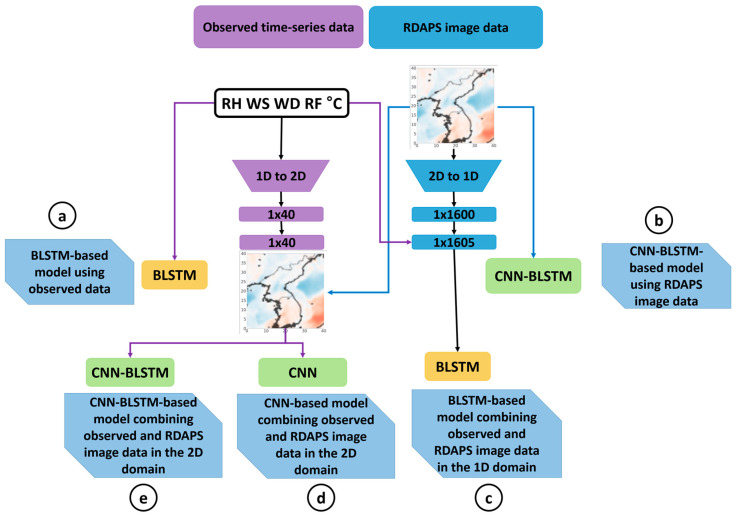
Block diagram of four different temperature prediction models for performance comparison with the proposed model: (**a**) a BLSTM-based model using observed data, (**b**) a CNN-BLSTM-based model using RDAPS image data, (**c**) a BLSTM-based model combining observed and RDAPS image data in the 1D domain, (**d**) a CNN-based model combining observed and RDAPS image data in the 2D domain, and (**e**) a CNN-BLSTM-based model combining observed and RDAPS image data in the 2D domain.

The proposed temperature prediction model was implemented in two different ways according to the way in which the attention mechanism was applied when combining the observed and RDAPS data. The prediction performance of the proposed model with or without attention was evaluated, and compared with those of five different temperature prediction models, as shown in Figure 5. The first was a BLSTM-based temperature prediction model using only observed data, which was identical to the model described in Section 3.2.1, and the second one was a CNN-BLSTM-based temperature model using only RDAPS data, which was identical to the model described in Section 3.2.2.

In addition, a BLSTM-based temperature prediction model was constructed by combining the observed data and RDAPS data in the 1D domain, which was similar to the approach introduced in [10]. In other words, each (40 × 40)-dimensional RDAPS image was flattened into (1 × 1600)-dimensional 1D data, and then this 1D data was augmented with the (1 × 5)-dimensional observed data, resulting in (1 × 1605)-dimensional data. These data were used as input features for the BLSTM-based model, where the network architecture was identical to the BLSTM-based model in Section 3.2.1.

Next, a CNN-based temperature prediction model was constructed by combining the observed data and RDAPS data in the 2D domain. To this end, the (1 × 5)-dimensional observed data were concatenated with the (40 × 40)-dimensional RDAPS data, producing (41 × 40) image data. After that, these data were input into a CNN that was identical to Figure 3a except for the dimension of the input features, where a dense layer was concatenated to the flattened layer for the future temperature predictions.

Lastly, a CNN-BLSTM-based temperature prediction model was constructed using 2D data, as proposed in [17]. Similar to the CNN-based temperature prediction model described above, the (1 × 5)-dimensional observed data were concatenated with (40 × 40)-dimensional RDAPS data, producing (41 × 40) image data. A CNN-BLSTM-based model was trained following the approach described in Section 3.2.2.

All of the models, including the proposed model and five other models, were trained and evaluated for each of seven different time periods, $t_{p}$. Specifically, $t_{p}$ was set to one of 6, 12, 24 (one-day), 72, 168 (seven-day), or 336 (14-day) h. As mentioned in Section 3.1, the observed data and RDAPS image data were collected over five years from 1 May 2011 to 31 December 2015, and they were split for training, validating, and evaluating the prediction models.

In this experiment, all neural network models were implemented using a deep learning package in Python 3.6.9 with Keras (version 2.3.1) using TensorFlow (version 2.2.0) [29]. The neural network weights of all the models were initialized using Xavier initialization [30], and the biases were all initialized to zero. Next, the mini-batchwise adaptive moment estimation (ADAM) optimization algorithm [31] was applied, with the minibatch size set to 64. The learning rate was set according to the ramp-up strategy [32,33], with the maximum learning rate reaching 0.001 after 50 epochs.

The training procedure was controlled by early stopping [34] for minimizing the validation error with 200 epochs. All the hyperparameters of the neural network models used in this paper, including the number of layers, number of kernels, and kernel size for CNN as well as the number of hidden layers and number of nodes for BLSTM, were selected from an exhaustive search over several possible combinations. The training and evaluation of the models were conducted on an Intel(R) Xeon(R) CPU E5-2623 v3 @ 3.00GHz workstation with an NVidia GTX 1080ti GPU.

### 4.1. Evaluation Metric

The performance of each of the temperature prediction models was evaluated using the IOA, R, RMSE, MAE, MBE, MNGE, and MNB, which are defined as follows:

(5) $$\mathrm{IOA}=1-\frac{\sum_{t=1}^{N}{\left(C_{t,pred}-C_{t,obs}\right)}^{2}}{\sum_{t=1}^{N}{\left(\left|C_{t,pred}-{\bar{C}}_{obs}\right|+\left|C_{t,obs}-{\bar{C}}_{obs}\right|\right)}^{2}}\;,$$

(6) $$R=\frac{\sum_{t=1}^{N}\left(C_{t,pred}-{\bar{C}}_{pred}\right)\left(C_{t,obs}-{\bar{C}}_{obs}\right)}{\sqrt{\sum_{t=1}^{N}{\left(C_{t,pred}-{\bar{C}}_{pred}\right)}^{2}}\sqrt{\sum_{t=1}^{N}{\left(C_{t,obs}-{\bar{C}}_{obs}\right)}^{2}}},$$

(7) $$\mathrm{RMSE}=\sqrt{\frac{1}{N}\sum_{t=1}^{N}{\left(C_{t,pred}-C_{t,obs}\right)}^{2}},$$

(8) $$\mathrm{MAE}=\frac{1}{N}\sum_{t=1}^{N}\left|C_{t,pred}-C_{t,obs}\right|,$$

(9) $$\;\mathrm{MBE}=\frac{1}{N}\sum_{t=1}^{N}\left(C_{t,pred}-C_{t,obs}\right),$$

(10) $$\mathrm{MNGE}=\frac{1}{N}\sum_{t=1}^{N}\frac{\left|C_{t,pred}-C_{t,obs}\right|}{C_{t,obs}}\times 100,$$

(11) $$\mathrm{MNB}=\frac{1}{N}\sum_{t=1}^{N}\frac{C_{t,pred}-C_{t,obs}}{C_{t,obs}}\times 100$$

where $C_{t,obs}$ and $C_{t,pred}$ are the observed and predicted temperature at time *t*, respectively, and *N* is the total number of evaluation data samples (*N* = 8760 in this paper). In addition, ${\bar{C}}_{pred}=\left(1/N\right)\sum_{t=1}^{N}C_{t,pred}$ and ${\bar{C}}_{obs}=\left(1/N\right)\sum_{t=1}^{N}C_{t,obs}$.

### 4.2. Performance Comparison

Table 2 shows the performance of the temperature prediction model applied to the observed data or RDAPS image data compared for the prediction of 6-, 12-, 24-, 72-, 168-, and 336-h future temperatures. As shown in Figure 5a,b, a BLSTM-based temperature prediction model was constructed using only observed data, while a CNN-BLSTM-based temperature model was made using only RDAPS data. The former was designed to deal with time-series data, and the latter was designed for image data.

To examine the advantage in the prediction performance of BLSTM over LSTM, an LSTM-based model was also constructed by replacing BLSTM with LSTM in the BLSTM-based model. As shown in the table, the prediction performance of both models decreased as the time period to be predicted increased. The RMSE, MAE, and MNGE were the highest and the IOA and R were the lowest for the 336-h prediction. Comparing the performances of the LSTM-based and BLSTM-based model showed that the BLSTM-based model achieved better performance than the LSTM-based one for all the prediction time periods, which motivated the use of BLSTM in this paper over LSTM.

It was also shown from the performance comparison between the BLSTM-based and CNN-BLSTM-based models that the evaluation metrics of the CNN-BLSTM-based model were always better than those of the BLSTM-based model for all of the prediction periods. This superiority arose because the CNN-BLSTM-based model provided spatial or regional information from the regional image, while the BLSTM-based model dealt with information localized to a specific area. This result indicated that it was better to use all information from various areas than to use only the observed data from a specific area.

Next, the performance of the temperature prediction models that were constructed by combining the observed and RDAPS image data were compared, where the combination was performed by converting the input data from 1D to 2D or vice versa, as shown in Figure 5c–e. In other words, a BLSTM-based temperature prediction model was constructed by combining the observed data and RDAPS data in the 1D domain, where each (40 × 40)-dimensional RDAPS image was flattened into (1 × 1600)-dimensional 1D data before combining. In addition, a CNN-based as well as a CNN-BLSTM-based temperature prediction model were constructed by concatenating the observed and RDAPS data in the 2D domain. The difference between the two models was that the BLSTM layer was followed by the CNN in the CNN-BLSTM-based model while the CNN-based model used the CNN outputs for the temperature prediction.

Table 3 compares the seven different evaluation measures between the BLSTM-based and CNN-BLSTM-based temperature prediction models for the 6-, 12-, 24-, 72-, 168-, and 336-h temperature predictions. As shown in the table, the CNN-based model achieved worse performance for all the prediction periods compared with the BLSTM-based and CNN-BLSTM-based models. When the prediction periods were shorter than 168 h (7 days), the performances of the BLSTM-based model were slightly better than those of the CNN-BLSTM-based model; however, the performance improvement was marginal. However, for longer predictions, such as 7- and 14-days, the 2D representation used in the CNN-BLSTM-based model was better than the 1D representation in the BLSTM-based model. This result implied that a suitable representation of the input data could improve the performance of the temperature prediction model.

The performance of the CNN-BLSTM-based model using only RDAPS image data, as shown in the second row of Table 2, was compared with that of the CNN-BLSTM-based model combining the observed and RDAPS image data in the 2D domain as shown in the third row of Table 3. The combination model in Table 3 was not always better than the single data model in Table 2, which implies that the architecture of a neural network should be carefully designed when combining different types of data.

Finally, Table 4 compares the performance of the proposed temperature prediction model without or with the dot product attention for the 6-, 12-, 24-, 72-, 168-, and 336-h temperature predictions. As shown in the table, the attention mechanism contributed to reducing the RMSE, MAE, and MNGE of the temperature predictions for short periods, such as 6, 12, and 24 h. However, the performance gain due to the attention mechanism was marginal for time periods longer than 24 h. This was because the attention mechanism could emphasize temperature features up to 24 h, and the input layers of neural networks used the observed and RDAPS image data of 24 h as input features.

The prediction performance produced by the combination method can be compared by examining the differences between the results in Table 3 and Table 4. The results of Table 3 corresponded to the combination of the observed time-series and RDAPS image data in the input level, while the combination was carried out in the feature representation level for the proposed model in Table 4. The proposed model with attention provided better performance according to all of the evaluation metrics compared with the CNN-BLSTM-based model combining the observed and RDAPS image data in the 2D domain, for prediction periods of up to 72 h.

However, their performances were comparable for longer prediction periods, such as 7 and 14 days. As mentioned in the previous paragraph, this was because the observed and RDAPS data of 24 h were used as input data for the neural networks, thus, the duration of input data should be increased to more than 24 h for longer time period predictions. By comparing the performance of the proposed model with those of the models in Table 2, it was demonstrated that the proposed model that combined both the observed and RDAPS image data was better in all performance measures for all prediction periods compared with the BLSTM-based and CNN-BLSTM-based models that used the observed data and RDAPS image data alone.

To evaluate the potential usefulness of the proposed model with an attention mechanism, an accuracy comparison between the proposed model and RDAPS was performed for 6, 12, 24, and 72 h temperature predictions from January 2014 to December 2014. Table 5 shows the prediction performance of the traditional UM model. As the UM forecasts up to 87 h, the prediction models up to 72 h were compared. The UM predicts weather information using various data, such as AWS, satellite, and radar collected by advanced observation technology and equipment. The prediction performance of UM is similar to the performance of the BLSTM-based model using the observed data. Based on this experiment using 1-year weather data, the proposed model with an attention mechanism achieved a lower RMSE in the 6, 12, 24, and 72 h predictions compared to the UM model.

Table 6 compares the model size of each of the seven different temperature prediction models developed in this paper. The BLSTM-based in Figure 5a had the smallest model size because it dealt with only observed data. On the other hand, the BLSTM-based model in Figure 5c increased the model size up to 21 MB because the observed and RDAPS image data were represented by 2D images. However, by representing RDAPS image data using CNN, the image data were compressed into lower dimensional data, thus, the CNN-BLSTM-based models in Figure 5b,e (while the former only used observed data and the latter used both observed and RDAPS image data) had smaller model sizes than the BLSTM-based model. Lastly, the proposed model increased in model size, because the proposed model concatenated the outputs of BLSTM from the observed data and those of CNN-BLSTM from the RDAPS image data. By incorporating the attention mechanism into the proposed model, the model size was increased up to 34 MB. We confirmed that the proposed model with an attention mechanism could infer the temperature prediction in real time.

**Figure 6:**
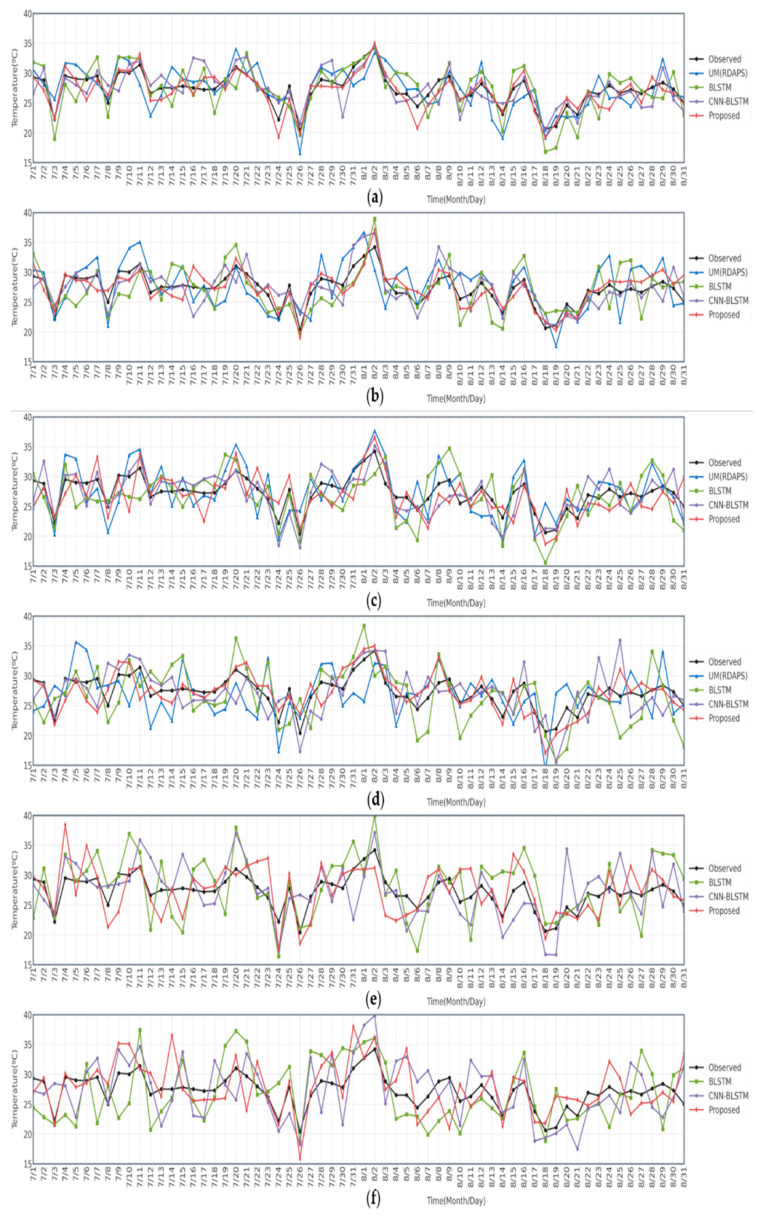
Daily plots of the observed and predicted temperature data during two months from July to August 2015 for the (**a**) 6-, (**b**) 12-, (**c**) 24-, (**d**) 72-, (**e**) 168-, and (**f**) 336-h predictions.

**Figure 7:**
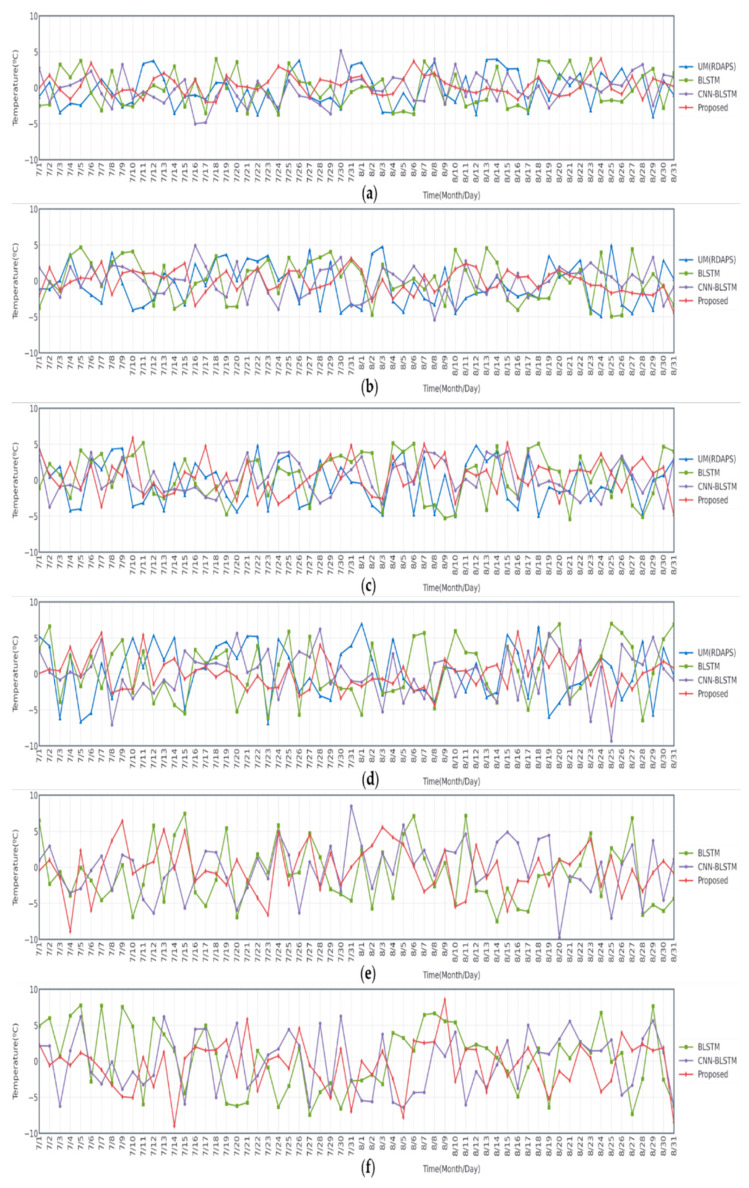
Daily plots of the differences between the observed and predicted temperature data during two months from July to August 2015 for the (**a**) 6-, (**b**) 12-, (**c**) 24-, (**d**) 72-, (**e**) 168-, and (**f**) 336-h predictions.

Finally, Figure 6 illustrates a time-series plot of the observed data and predicted temperature data during two months from July to August 2015 for the 6-, 12-, 24-, 72-, 168-, and 336-h future predictions. In this figure, three different models, the BLSTM-based model using observed data, the CNN-BLSTM-based model using RDAPS image data, and the proposed model with an attention mechanism using both the observed and RDAPS image data. The temperature data predicted by UM (RDAPS) were added for the 6-, 12-, 24-, and 72-h predictions. In parallel, the differences between the observed data and the predicted temperature data are illustrated in Figure 7. As shown in the figures, among all the prediction models, the temperatures predicted by the proposed model with an attention mechanism were, on average, the closest to the observed temperature data. The proposed model had the lowest average MAE measured from July to August 2015.

## 5. Conclusions

This paper proposed a deep neural network-based temperature prediction model using both time-series observed weather data and RDAPS image data. Instead of combining these different types of data at the input feature level, the feature representation part of the proposed model applied two different neural networks to combine the different types of input data. A BLSTM neural network and a CNN-BLSTM neural network were used to handle the time-series observed data and the RDAPS image data, respectively. Then, two feature maps, one from the BLSTM and one from the CNN-BLSTM, were concatenated by adding them or by applying an attention mechanism to emphasize the correlation of temperature between the observed and the numerical forecast image data.

The performance of the proposed temperature prediction model was evaluated by seven different objective measures—IOA, R, RMSE, MAE, MBE, MNGE, and MNB—and compared with those of a temperature prediction model using either the observed data or RDAPS image data. The proposed model combining both observed and RDAPS image data was better in all performance measures for all prediction periods compared with the BLSTM-based and CNN-BLSTM-based models using the observed data and RDAPS image data alone, respectively.

Two different temperature prediction models were constructed, combining the observed data and RDAPS image data in the time-series and image domain, respectively. The proposed model with attention produced better performance in all of the evaluation metrics compared with the CNN-BLSTM-based model combining the observed and RDAPS image data in the 2D domain, when the prediction periods were up to 72 h. Their performances were comparable for longer prediction periods, such as 7 and 14 days. This result might be because the observed and RDAPS data of only 24 h were used as input data for the neural networks, thus, the duration of the input data should be increased to more than 24 h for longer time period predictions.

In future work, to further improve the performance of the proposed temperature prediction model for relatively long time periods, such as 7 and 14 days, the proposed model could be extended using time-series and RDAPS image data for more than 24 h. In addition, the time-variant fuzzy information technique in [35] and the super-resolution generative adversarial network (SRGAN) in [36] could be incorporated into the proposed model for better representation of time-series data representation and RDAPS image data, respectively. The CNN layer used in this paper could be replaced with recently developed convolutional networks, such as a residual convolutional neural network (ResNet) [37] or dense convolutional neural network (DenseNet) [38]. Finally, since the proposed model is applicable not to only temperature but also to other weather factors, this research can be extended to predict other weather factors.

## Figures and Tables

**Table 1:** Comparison of three numerical weather prediction (NWP) models of Korean Meteorological Agency (KMA).

Model	Horizontal Resolution (km)	Number of Vertical Layers (Top Height)	Forecast Period (H)	Forecast Cycle (H)	Horizontal Grid (East to West, North to South Direction)
GDAPS	25	70 (80 km)	87 288	3 6	1024 × 769 (25 km from 0° E, 0° S)
RDAPS	12	70 (80 km)	87	3	491 × 419 (12 km from 101.577° E, 12.217° S)
LDAPS	1.5	70 (40 km)	36	1	602 × 781 (1.5 km from 121.834° E, 32.257° N)

**Table 2:** Performance comparison of the seven different evaluation measures between the LSTM-based, BLSTM-based, and CNN-BLSTM-based temperature prediction models applied to the observed and RDAPS image data for the 6-, 12-, 24-, 72-, 168-, and 336-h temperature predictions.

Model	Time (H)	Evaluation Metric						
		IOA	R	RMSE	MAE (°C)	MBE (°C)	MNGE (%)	MNB (%)
LSTM-based model using observed data	6	0.98	0.97	2.40	1.76	0.60	0.61	0.21
	12	0.98	0.96	2.81	2.08	−0.15	0.73	−0.04
	24	0.97	0.94	3.24	2.43	0.14	0.85	0.06
	72	0.96	0.92	3.88	2.94	−0.42	1.02	−0.14
	168	0.95	0.91	4.24	3.25	−0.37	1.14	−0.15
	336	0.94	0.89	4.48	3.49	−0.53	1.22	−0.17
BLSTM-based model using observed data	6	0.99	0.98	2.38	1.75	0.61	0.61	0.22
	12	0.98	0.97	2.70	1.96	0.32	0.70	0.11
	24	0.98	0.96	3.05	2.28	0.45	0.81	0.17
	72	0.96	0.93	3.83	2.94	−0.60	1.02	−0.20
	168	0.96	0.92	4.08	3.14	−0.68	1.11	−0.23
	336	0.95	0.91	4.42	3.40	−0.92	1.19	−0.31
CNN-BLSTM-based model using RDAPS image data	6	0.99	0.98	2.17	1.68	−0.02	0.57	0.00
	12	0.99	0.98	2.23	1.71	−0.09	0.60	−0.03
	24	0.99	0.97	2.47	1.83	0.04	0.65	0.02
	72	0.97	0.95	3.44	2.49	0.56	0.91	0.20
	168	0.97	0.93	3.72	2.86	−0.43	1.02	−0.14
	336	0.96	0.92	3.98	3.09	−0.40	1.09	−0.13

**Table 3:** Performance comparison of the seven different evaluation measures between the BLSTM-based, CNN-based, and CNN-BLSTM-based temperature prediction models applied to the combination of the observed and RDAPS image data for 6-, 12-, 24-, 72-, 168-, and 336-h temperature predictions in the 1D domain and 2D domain.

Model	Time (H)	Evaluation Metric						
		IOA	R	RMSE	MAE (°C)	MBE (°C)	MNGE (%)	MNB (%)
BLSTM-based model combining observed and RDAPS image data in the 1D domain	6	0.99	0.98	2.11	1.59	0.03	0.56	0.02
	12	0.99	0.98	2.21	1.68	0.84	0.59	0.30
	24	0.99	0.98	2.35	1.77	0.52	0.62	0.19
	72	0.97	0.95	3.31	2.53	−0.67	0.89	−0.23
	168	0.96	0.93	3.78	2.95	0.34	1.04	0.13
	336	0.94	0.89	4.68	3.75	0.49	1.32	0.19
CNN-based model combining observed and RDAPS image data in the 2D domain	6	0.97	0.95	3.13	2.45	−0.24	0.85	−0.07
	12	0.97	0.96	3.05	2.41	1.54	0.84	0.54
	24	0.97	0.95	3.15	2.45	0.62	0.86	0.22
	72	0.95	0.92	4.23	3.34	1.56	1.17	0.55
	168	0.95	0.91	4.19	3.27	0.53	1.15	0.20
	336	0.93	0.89	4.77	3.78	−1.41	1.32	−0.47
CNN-BLSTM-based model combining observed and RDAPS image data in the 2D domain	6	0.99	0.98	2.23	1.72	0.50	0.60	0.18
	12	0.99	0.98	2.16	1.68	−0.14	0.59	−0.04
	24	0.99	0.97	2.42	1.89	0.52	0.66	−0.17
	72	0.97	0.95	3.31	2.49	0.21	0.88	0.08
	168	0.96	0.94	3.67	2.90	−0.69	1.02	−0.23
	336	0.96	0.92	3.91	2.99	0.19	1.06	0.08

**Table 4:** Performance comparison of the seven different evaluation measures of the proposed temperature prediction model without and with an attention mechanism for 6-, 12-, 24-, 72-, 168-, and 336-h temperature predictions.

Model	Time (H)	Evaluation Metric						
		IOA	R	RMSE	MAE (°C)	MBE (°C)	MNGE (%)	MNB (%)
Proposed model without attention mechanism	6	0.99	0.98	1.93	1.37	−0.06	0.47	−0.02
	12	0.99	0.98	2.12	1.55	0.20	0.54	0.07
	24	0.99	0.98	2.34	1.71	0.42	0.60	0.15
	72	0.97	0.95	3.17	2.41	−0.50	0.85	−0.17
	168	0.97	0.93	3.71	2.87	0.21	0.99	0.08
	336	0.96	0.92	3.90	3.00	0.01	1.06	0.02
Proposed model with attention mechanism	6	0.99	0.98	1.90	1.34	0.02	0.47	0.01
	12	0.99	0.98	1.98	1.46	0.30	0.51	0.10
	24	0.99	0.98	2.27	1.66	0.31	0.58	0.12
	72	0.97	0.95	3.26	2.43	0.17	0.86	0.07
	168	0.97	0.93	3.71	2.81	−0.46	0.99	−0.15
	336	0.96	0.93	3.83	2.96	0.14	1.05	0.06

**Table 5:** Performance of the seven different evaluation measures of the traditional method UM (RDAPS) for 6-, 12-, 24-, and 72-h temperature predictions.

Model	Time (H)	Evaluation Metric						
		IOA	R	RMSE	MAE (°C)	MBE (°C)	MNGE (%)	MNB (%)
UM(RDAPS)	6	0.98	0.97	2.38	1.75	0.60	0.61	0.21
	12	0.98	0.96	2.76	2.04	−0.15	0.71	−0.05
	24	0.97	0.95	3.01	2.28	0.11	0.80	0.04
	72	0.96	0.93	3.74	2.81	−0.24	0.99	−0.07

**Table 6:** Model size comparison of different temperature prediction models performed for the prediction time of 6 h.

Model	BLSTM (Figure 5a)	CNN-BLSTM (Figure 5b)	BLSTM (Figure 5c)	CNN (Figure 5d)	CNN-BLSTM (Figure 5e)	Proposed w/o Attention	Proposed with Attention
Model size	3 MB	17 MB	21 MB	7 MB	17 MB	26 MB	34 MB

## Data Availability

Publicly available datasets were analyzed in this study. This data can be found here: https://data.kma.go.kr/data/grnd/selectAwsRltmList.do?pgmNo=56 and https://data.kma.go.kr/data/rmt/rmtList.do?code=312&pgmNo=64.
